# Diagnostic and prognostic relevance of plain radiographs for periprosthetic joint infections of the hip: a literature review

**DOI:** 10.1186/s40001-024-01891-8

**Published:** 2024-06-08

**Authors:** Ulf Krister Hofmann, Georgios Eleftherakis, Filippo Migliorini, Bernd Fink, Moritz Mederake

**Affiliations:** 1 Department of Orthopaedic, Trauma, and Reconstructive Surgery, RWTH University Medical Centre, Pauwelsstraße 30, 52074 Aachen, Germany; 2 Department of Orthopaedic Surgery, University Hospital of Tübingen, 72076 Tübingen, Germany; 3 Department of Orthopedics and Trauma Surgery, Academic Hospital of Bolzano (SABES-ASDAA), Teaching Hospital of the Paracelsus Medical University, 39100 Bolzano, Italy; 4 Department of Arthroplasty and Revision Arthroplasty, Orthopaedic Clinic Markgröningen GmbH, Kurt-Lindemann-Weg 10, 71706 Markgröningen, Germany; 5 Orthopaedic Department, University-Hospital Hamburg-Eppendorf, Martinistrasse 52, 20246 Hamburg, Germany; 6 Department of Trauma and Reconstructive Surgery, BG Klinik, University of Tübingen, 72076 Tübingen, Germany

**Keywords:** Radiography, Periprosthetic joint infection, Hip arthroplasty

## Abstract

Conventional radiography is regularly used to evaluate complications after total hip arthroplasty. In various recent consensus meetings, however, plain radiographs of a potentially infected hip joint have been judged as being only relevant to exclude diagnoses other than infection. Solid data on radiographic presentations of periprosthetic joint infection (PJI) are scarce. As a result, the prognostic value of radiological features in low-grade PJI remains uncertain. The present review article aims to present an overview of the available literature and to develop ideas on future perspectives to define the diagnostic possibilities of radiography in PJIs of the hip. The primary outcome of interest of this systematic review was the radiologic presentation of periprosthetic joint infections of the hip. As secondary outcome of interest served the sensitivity and specificity of the radiologic presentation of periprosthetic joint infections. Of the included articles, 26 were reviews, essays, or case reports and only 18 were clinical studies. Typical radiologic abnormalities of PJI were a periosteal reaction, a wide band of radiolucency at the cement–bone or metal–bone interface, patchy osteolysis, implant loosening, bone resorption around the implant, and transcortical sinus tracts. The frequency of their occurrence is still inadequately defined. A deeper understanding of the underlying causes and the relation between microorganisms to radiologic abnormalities can probably help clinicians in the future to diagnose a PJI. This is why further research shall focus on the radiographic features of PJI.

## Introduction

Conventional radiography is regularly used to evaluate joint prostheses after implantation and during follow-up, as X-rays can detect potential abnormalities involving both the implant and the surrounding bone. Such abnormalities could be for example periprosthetic fracture, dislocation, osteolysis due to third body wear, or sinking of the shaft. One of the major complications after arthroplasty is periprosthetic joint infection (PJI). In various recent consensus meetings, however, plain radiographs of a potentially infected hip joint have been judged as being only relevant to exclude diagnoses other than infection [1–3]. In the consensus statement published by Romano and colleagues in 2020, for example, the diagnostic performance of conventional radiography in detecting PJI would be very low.

Furthermore, conventional radiography would show demineralization only when more than 30–50% of bone mass has been lost. Abnormalities of bone around the implant would usually be non-specific for infection. In addition, up to 50% of conventional X*-*ray exams would give negative results [1]. In another recent consensus statement published by Signore et al. [2, 3], the authors state that “Regarding PJI, conventional radiography often yields normal results or may detect non-specific signs of soft-tissue swelling. Serial plain radiography has been reported to have a sensitivity of 14% and specificity of 70% in detecting implant-associated infections [4]. Radiographic signs that may reveal PJI with high specificity are gas formation and active, immature periostitis. Radiographic signs with low specificity include soft-tissue swelling, periprosthetic lucency, and component loosening. However, differentiation between septic and aseptic periprosthetic lucency and component loosening is almost impossible in conventional radiography. Also, these signs are visible only when almost 30% of the bone mass has been lost; thus, 50% of radiographs remain normal despite the presence of infection”. These consensus statements are, however, only based on three references [5–7].

Most of the studies to which these statements relate date to the late 80 s and early 90 s [5, 7, 8]. The foundations of the data referenced here are also quite weak, such as in the study from Tigges et al., [7] with 20 confirmed infected hip arthroplasties, or Lyons et al. [5] 50 painful hip arthroplasties. The largest series presented was from Thoren and Hallin in 1989 [8], where the authors analysed 102 hip revisions. Of these, however, only 47 were infected and the prostheses analysed were original Charnley prostheses which possessed a 22.225-mm head in ultra-high molecular weight polyethylene (UHMW PE) in an all-cemented technique and a metal-on PE bearing. The arthroplasty landscape has, however, largely changed since then. Today's arthroplasties often contain an uncemented cup and a ceramic head. Depending on the country the stem is often uncemented ranging from 37% in the National Joint Registry in Great Britain to 78% in the German Arthroplasty Registry in Germany [9]. Uncemented stems usually consist of titanium which altogether changes the immunogenicity of the wear particles generated. Moreover, the annual number of patients treated with arthroplasties has multiplied and as such the surgical technique and postoperative rehabilitation protocols have been optimized and largely standardized. In addition, life expectancy has increased with people in old age having multiple comorbidities in addition to joint replacement. This supposedly changes the spectrum of bacteria responsible for infections. While we are quite effective in treating acute PJIs, the successful management of low-grade infections is still a challenge. This includes reliable, sensitive and specific diagnostics of such low-grade PJIs. Nevertheless, substantial progress has been made in the diagnosis of such an event with improvements in the histopathological analysis of the periprosthetic membrane [10, 11], the advent of PCR analyses [12], and synovial fluid analysis including the analysis of PMNs, alpha-defensin or leukocyte esterase-levels [13–16]. Other newer markers are presently under investigation, such as pentraxin-3 [17], calprotectin [18], or myeloperoxidase [19]. Of note, thresholds for leukocytes in low-grade PJIs have been constantly lowered over the past decades ranging now—depending on the joint—between 1000 and 2000 leukocytes/µl only [20]. Cultivation techniques for bacteria have also been more and more standardized. We can assume that the overall sensitivity and specificity have increased over the past 30 years. Similarly, the technique of acquiring radiographs has also improved with the widespread introduction of digital radiography. The digital data set allows for post-imaging optimization of each X-ray to visualize structures that were difficult to discern in traditional images intended for a good bone contrast only. Nevertheless, solid data on radiographic presentations of PJI are scarce. As a result, the prognostic value of radiological features in low-grade PJI remains uncertain. The present review article aims to present an overview of the available literature and to develop ideas on future perspectives to define the diagnostic possibilities of radiography in PJIs of the hip.

## Materials and methods

### Eligibility criteria

All published articles related to the radiographic presentation of PJI of the hip were accessed. Only articles available in English, French, Spanish or German were eligible. Original studies with a level of evidence of I to IV according to the Oxford Centre of Evidence-Based Medicine [21] plus review articles and essays were considered.

### Search strategy

This systematic review was conducted according to the Preferred Reporting Items for Systematic Reviews and Meta-Analyses: the 2020 PRISMA statement [22]. On July 24, 2023, PubMed and Web of Science were accessed. The following keywords were used: "periprosthetic joint infection hip radiograph"; "Prosthesis-Related Infections"[Mesh] AND "Radiography"[Mesh]) AND "Hip"[Mesh]; "Radiography"[Mesh]) AND "Hip"[Mesh]) AND "Infections"[Mesh]; "radiography" "hip arthroplasty" "infection"; "x-ray" "hip arthroplasty" "infection"; "x-ray" "periprosthetic joint infection" "hip"; "periprosthetic joint infection" "hip" "radiograph"; "heterotopic ossification" "hip arthroplasty" "infection"; "heterotopic ossification" "hip" "periprosthetic joint infection". No filters were applied.

### Selection and data collection

Three authors (UKH; MM; GE) independently performed the database search. All the resulting titles were screened and, if suitable, the abstract was accessed. The full text of the abstracts, which matched the topic, was accessed. A cross-reference of the bibliography of the full-text articles was also screened for inclusion. If the full text was not accessible or available, the article was not considered for inclusion. Disagreements were debated before final inclusion into the study.

### Data items and outcome of interest

The following data at baseline were extracted: author, year of publication and journal, PMID/PCMID/DOI, type of analysis performed, country of origin, main study outline, number of patients used for the relevant statements, and statements made regarding radiography and PJI of the hip. A suitable level of evidence was attributed to each study. The primary outcome of interest was the radiologic presentation of PJI of the hip. As secondary outcome of interest served the sensitivity and specificity of the described image characteristics.

## Results

### Study selection

**Fig. 1:**
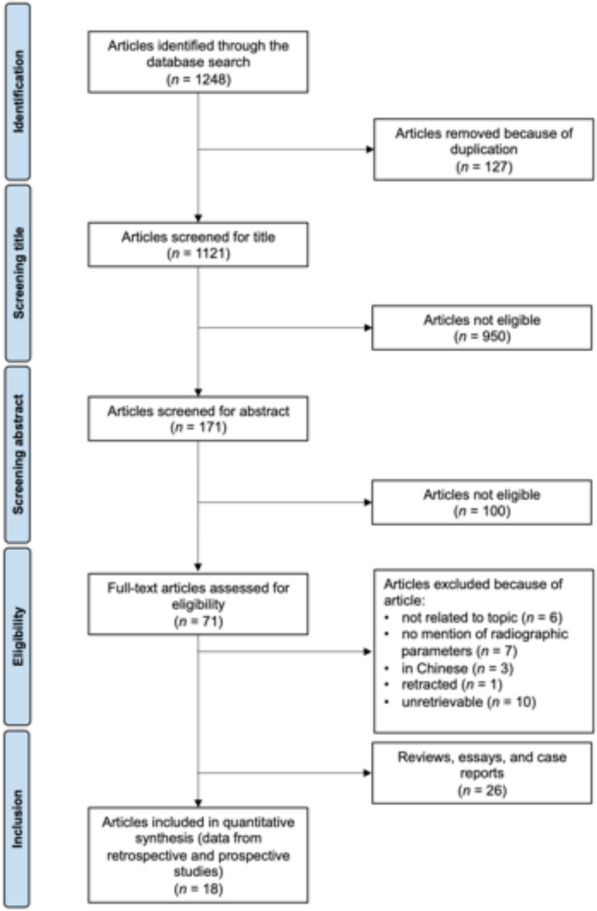
Flowchart of the literature search

The literature search resulted in 1248 articles (Fig. 1). After the removal of duplicates, 1121 articles were screened. Having screened titles and abstracts, the original manuscript was accessed for 71 studies. Of these 44 finally met the inclusion criteria, the other publications were either in Chinese (*n =* 3), not retrievable (*n =* 10), retracted (*n =* 1), were not related to the research question (*n =* 6), or did not directly report radiographic parameters (*n =* 7). Of the finally included articles, 26 were reviews, essays, or case reports. Only 18 publications were clinical studies with a level of evidence between IV and II.

### Review summary

Summarizing the statements provided in the review articles, essays, and case reports (Table 1), a few commonly reported traits can be made out: claimed radiographic signs of infection are periosteal reaction [23–33], formation of lamellae [23], focal osteolysis or bone destruction [7, 23, 24, 26, 28, 31, 33–38], a wide radiolucent zone [23, 32, 36, 39], signs of loosening in a previously well-fixed implant [24, 27, 28, 32, 34], heterotopic bone formation [28, 40], mottling [31], an intracortical sinus tract [24, 28], periprosthetic fractures [27], adjacent soft-tissue collection [29, 30, 32], and rapid disease progression [25]. Aseptic loosening tends to produce uniform radiolucency, whereas particle disease produces multifocal radiolucencies related to localized osteolysis. Infection can produce either of these patterns [41, 42] with an osteolysis of > 2 mm being indicative of infection [41]. The radiographic presentation thereby seems to be a function of time, with early infections presenting without radiological features and late infections presenting with inflammatory and reactive osteoproliferative changes of the bone [34, 43]. These signs are only present in a subpopulation of all PJI and thus have a low sensitivity while having a reasonable specificity.

**Table 1:** Review articles, essays, and case reports addressing the aspect of radiographic presentation of infected total hip arthroplasties

Author and year	Journal	Level of evidence	Study design	Country	Main article content	Sample size on which statement is based	Main radiographic statements with respect to PJI
Awan et al., 2013 [41]	Can Assoc Radiol J	Narrative review	Review	USA	Narrative review on imaging evaluation of complications of hip arthroplasty	–	Aseptic loosening tends to produce uniform radiolucency, whereas particle disease produces multifocal radiolucencies related to localized osteolysis. Infection can produce either of these patterns. Multifocal osteolysis around the prosthesis (> 2mm or more) raises concern for infection. This phenomenon is, however, not always present
Breitenseher et al., 2002 [23]	Radiologe	V	Essay	Germany	Essay on radiographic presentation of hip arthroplasties. A radiolucent zone with adjacent sclerosis is acceptable in cemented components if smaller than 2 mm and only around parts of the implant. If more than 50% of the implant are affected, loosening should be considered	–	Radiographic signs of infection are periosteal reaction, formation of lamellae, osteolysis, and wide radiolucent zone
Bültmann et Dihlmann, 1973 [43]	Fortschr Geb Röntgenstr Nuklearmed	V	Essay	Germany	Essay on radiographic presentation of complications after hip arthroplasty	–	Prosthesis loosening in a metal-on poly articulation are considered suggestive for infection. Early infections present without radiological features, late infections with inflammatory and reactive osteoproliferative changes of the bone
Chang et al., 2015 [42]	Semin Musculoskelet Radiol	Narrative review	Narrative review	USA	Describes the radiographic presentation of hip implants in a regular and pathological context	–	Radiographic findings in infection are often similar to those seen in aseptic loosening or particle disease
Enayatollahi et Parvizi, 2015 [24]	Hip Int	Narrative review	Narrative review	USA	Review on the diagnosis of infected total hip arthroplasty	–	Signs on radiographs suggestive for PJI: • focal osteolysis or bone destruction • signs of loosening in a previously well-fixed component • subperiosteal reactions • intracortical sinus tract
Enge Júnior et al., 2020 [25]	Radiol Bras	V	Essay	Brazil	Describes the radiological presentation of main complications after hip arthroplasty	–	The presence of femoral periosteal reaction or rapidly progressive disease is indicative of septic loosening
Fritz et al., 2013 [26]	Semin Musculoskelet Radiol	Narrative review	Narrative review	USA	Describes the radiological presentation of main complications after hip arthroplasty	–	Radiographs are insensitive for the detection of implant infection. Periosteal reaction and osteolysis can be seen in the setting of infection but are not specific because similar findings can be seen in aseptic loosening and stress reaction
Hargunani et al., 2016 [34]	Can Assoc Radiol J	V	Essay	UK	Describes the radiological presentation of main complications after hip arthroplasty	–	Plain radiographs may be normal in the case of early infection. In late infection often loosening or bone destruction is observed. To discriminate between aseptic and septic loosening, comparison with prior films is important
Khodarahmi et al., 2017 [64]	Semin Musculoskelet Radiol	V	Essay	USA	Describes the MRI–appearance of prosthetic hip and its possible complications		MRI findings of periprosthetic joint infections are synovial enhancement and oedema, periarticular oedema, joint effusion, extracapsular collections and sinus tracts to the skin surface, bone destruction, and reactive lymphadenopathy
Kinoshita et al., 2021 [40]	Case Rep Orthop	V	Case report	Japan	Case presentation of a 78-year-old male with severe heterotopic ossification after total hip arthroplasty caused by coagulase-negative staphylococci	1	The patient developed severe heterotopic ossifications (Brooker IV). Coagulase-negative staphylococci were detected in the revision surgery
Koutserimpas et al., 2022 [65]	Diagnostics (Basel)	Review	Systematic review	Greece	A review of published fungal hip periprosthetic joint infections was conducted. Information regarding demographics, causative fungus, treatment and the infection outcome was recorded in 89 patients. Taken together, the authors describe a challenging diagnostic and treatment process	89	In a series of fungal periprosthetic joint infections in 20.2% of cases plain X-ray or CT scans were performed. Low sensitivity as well as specificity for definite PJI diagnosis is reported, as these changes are also observed with aseptic processes
Lee et al., 2015 [27]	J Orthop Translat	Narrative review	Narrative review	South Korea / USA	Review on the diagnostic options as well as the management and results of periprosthetic joint infections	–	Regarding plain radiographs the authors report a study with 50% of normal radiographs in a known periprosthetic joint infection cohort. Findings in periprosthetic joint infection are fracture, loosening and periosteal reactions
Lohmann et al., 2017 [35]	EFORT Open Rev	Narrative review	Narrative review	Germany	Review on radiographic periprosthetic assessment	–	In periprosthetic joint infection, radiographs are often normal. Osteolysis or bone resorption, however, especially seen less than five years postoperatively should not be misinterpreted as wear but may be indicative for infection
Love et al., 2009 [66]	Semin Nucl Med 2009	Narrative review	Narrative review	USA	Review on radiographic periprosthetic assessment with a focus on different radionuclide imaging methods and their diagnostic value	–	Plain radiographs have a lack of sensitivity and specificity and cross sectional imaging modalities can be limited by hardware induced artefacts
Lüdemann et al., 2015 [28]	Oper Orthop Traumatol	Narrative review	Narrative review	Germany	Review on the diagnosis on periprosthetic joint infections of the hip	–	Focal osteopenia and loosening provide indications of the extent of the infection. Further conventional radiological evidence of the presence of an infection can include periosteal reaction, subperiosteal new bone formation, transcortical fistula, and periarticular calcifications
Miller, 2012 [29]	Eur J Radiol	Narrative review	Narrative review	USA	Review on radiographic periprosthetic assessment	–	The presence of periosteal reaction in plain radiographs or computed tomography is highly predictive of infection. Furthermore, adjacent soft-tissue collection is also predictive
Mulcahy et al., 2012 [30]	AJR Am J Roentgenol	Narrative review	Narrative review	USA	Review on radiographic periprosthetic assessment of the hip	–	Femoral periosteal reaction or an adjacent soft-tissue collection is highly suggestive for hip periprosthetic joint infection. Previous radiographs are usually necessary for comparison
Mullins et al., 1974 [31]	Am J Roentgenol Radium Ther Nucl Med	Narrative review	Narrative review	USA	Radiographic evaluation of total hip arthroplasty complications	–	Radiographic signs of infection are mottling of bone, bone resorption and periosteal reactions. There are also cases of infection without radiologic abnormalities
Mushtaq et al., 2016 [36]	Front Surg	Narrative review	Narrative review	UK	Radiographic evaluation of failing total hip arthroplasties	–	Osteolysis and radiolucency is indicative for infection. However, a single radiograph is not able to differentiate between infection and other complications. In computed tomography, soft-tissue findings are highly indicative for infection (100% sensitivity, 87% specificity)
Parvizi et al., 2016 [32]	Orthop Clin North Am	Narrative review	Narrative review	USA	Review on the diagnosis of periprosthetic joint infections of the hip and the knee	–	Common radiographic findings of periprosthetic joint infections are cement fracture, subperiosteal reactions and rapid loosening. Furthermore, radiolucencies of > 2mm at the metal–bone–interface are indicative for infection
Roth et al., 2012 [67]	Radiographics	V	Essay	USA	Describes the radiological appearance of components, fixation and complications that can be seen on CT scans after hip arthroplasty	–	Osteolysis in CT scans can be an indicator for a PJI. Periosteal bone reactions in CT scans has a 100% specificity and a 16% sensitivity for infections. Soft-tissue findings as joint effusions or fluid collections can increase the sensitivity for infections
Segal and Krauss, 2007 [39]	J Arthroplasty	IV	Case report	USA	Describes the treatment of a PJI caused by Bacillus Calmette–Guerin after intravesical BCG application for transitional cell bladder carcinoma treatment	1	In connection with the periprosthetic joint infection caused by the BCG, radiographs showed radiolucencies in the periprosthetic area
Song et al., 2021 [37]	BMC Infect Dis	IV	Case report	China	Described the first case of periprosthetic joint infection caused by Clostridium difficile in China	1	On the radiography, a massive proximal femoral bone defect was visible, which also led to a shortened leg
Thejeel and Endo, 2022 [33]	Clin Imaging	Narrative review	Narrative review	USA	Reviews the different radiological signs of dislocation, infection, loosening and soft-tissue injury after total hip arthroplasty	–	Rapid progressive osteolysis and irregular periosteal reactions are specific signs for periprosthetic joint infections but not sensitive. CT and ultrasound showing irregular periostitis and extra-articular fluid collection may increase the specificity for infections. Lamellated synovium in MRI shows sensitivities of 80–88% and specificities of 84–92% for periprosthetic joint infection. Additional signs for periprosthetic joint infection in MRI investigations can be periosteal reaction, capsular and intramuscular oedema
Tigges et al., 1994 [57]	AJR Am J Roentgenol	V	Essay	USA	Describes the radiographic presentation of aseptic and septic loosening, stress shielding and aggressive granulomatosis after total hip arthroplasty	–	Plain radiographic findings are usually not enough to establish the diagnosis of periprosthetic joint infection. Suggestive radiographic signs for infection can be focal lucencies
Van Odijk et al., 1974 [38]	J Belge Radiol	V	Essay	Belgium	Essay on radiographic presentation of complications after hip arthroplasties	–	Radiographic signs of infection are osteolytic areas with bone erosion around the components of the prosthesis

### Original data

Looking at the original data (Table 2), the numbers on which statements are based are generally low ranging from 2 to 50 with a median of 15. The discrepancies in the reported data are, however, extreme: radiographic abnormalities are visible in all cases of PJI of the hip [44–47]. Other authors only report very low incidences of such findings [48–50]. Related features are periosteal reaction [5, 45], a radiolucent zone [7, 45], sinking of the prosthesis or loosening [45, 49–51], periprosthetic osteolysis or scalloped endosteal bone resorption [5, 7, 38, 50, 52], scalloping [45], and in cemented shafts cement mantle irregularities [51]. The first changes appear to be visible 3 months after the onset of symptoms [45]. The absence of periprosthetic osteolysis was reported to be predictive of aseptic loosening [52]. Lyons et al., reported a sensitivity of 47% and a specificity of 96% for scalloped bone resorption whereas laminated periosteal new bone had only a 25% sensitivity with, however, also a 92% specificity (*n =* 16) [5].

**Table 2:** Original studies addressing the aspect of radiographic presentation of infected total hip arthroplasties

Author and year	Journal	Level of evidence	Study design	Country	Main article content	Sample size on which statement is based	Main radiographic statements with respect to PJI
Barrack et al., 1993 [44]	J Bone Joint Surg Am	II	Retrospective study	USA	The value of aspiration of the hip joint before revision total hip arthroplasty. 270 cases were reviewed with joint aspiration prior to revision surgery with a documented follow-up of at least two years. 2% were actually diagnosed as infected. No patient in that series had a true-positive result in the absence of clinical or radiographic signs of infection	6	All 6 infected hips had radiographic findings compatible with infection: 5 × S. epidermidis: periostitis (3x), focal lysis (1x), diffuse lysis with endosteal scalloping (1x), heterotopic ossification (2x) 1 × Strept. sanguinis: focal lysis
Bergström et al., 1974 [45]	Clin Orthop Relat Res	II	Retrospective study	Sweden	In a retrospective collective having received total hip arthroplasty (*n =* 283) radiographic presentation of postoperative infections was evaluated in patients with persistent pain (*n =* 14) after surgery in a follow-up exceeding 1 year. 2 cases with deep wound infection postoperatively, 2 cases with late fistulation, 10 cases with continuous pain, elevated erythrocyte sedimentation rate and evidence of infection by plasma-electrophoresis. Radiographic evaluation was performed 1 week, 6 weeks, 3 months, 6 months, 1 year and 1 1/2 years after surgery	14	One year after surgery, all infected hips showed clear radiographic abnormalities. No findings were detected at 6 weeks after surgery Main finding were a periosteal reaction, radiolucent zone, sinking of the prosthesis, and scalloping, which in most cases occurred 3 months after onset of symptoms. One year postoperatively 8 patients presented all three features in radiographs. The most prevalent finding was that of periosteal reaction. 1 patient had strong formation of heterotopic ossification. In uninfected patients, half of the cemented hips showed a radiolucent line (< 2 mm) around the femoral component, which, however, never increased beyond 6 months postoperatively
Burastero et al., 2020 [53]	PLoS One	II	Retrospective study	Italy	Analyses a collective of two-stage hip arthroplasty septic revision with a focus on clinical and functional outcome, survival, mortality, eradication, reinfection and re-revision rates	148	Heterotopic ossifications were observed in 17 patients after two-stage septic hip revision (12 Brooker grade II and 5 Brooker grade III)
Isern-Kebschull et al., 2020 [52]	Skeletal Radiol	II	Retrospective study	Spain	Analyses the role of computer tomography in the discrimination of aseptic and septic loosening of a hip arthroplasty (*n =* 96). Infection was confirmed in 35 patients upon positive microbiologic culture	35	Associated roentgenologic features with infection were periprosthetic osteolysis (*n =* 23) without expansile periosteal reaction and with expansile periosteal reaction (*n =* 8). The absence of periprosthetic osteolysis was predictive of aseptic loosening. Expansile periosteal reaction was mostly observed in a separate granulomatosis group. Heterotopic bone formation occurred in all groups (infection, aseptic loosening, granulomatous reaction and mixed granulomatous and infection)
Isern-Kebschull et al., 2019 [55]	J Arthroplasty	II	Retrospective study	Spain	Uses the same study and collective as in [52]	35	In the diagnosis of a periprosthetic joint infection of the hip periarticular calcifications are reported with a sensitivity of 20.0 (8.4–36.9), a specificity of 78.7 (66.3–88.1), a positive predictive value of 35.0 (15.4–59.2), and a negative predictive value of 63.2 (51.3–73.9)
Itasaka et al., 2001 [68]	J Orthop Sci	II	Retrospective study	Japan	Analyses the accuracy of diagnostic tests including radiographs in a collective of 48 patients receiving revision of total hip arthroplasty. Infection was diagnosed on positive culture and histopathology	6	No association was found between an infected hip arthroplasty and the Gustilo and Pasternak classification [69]
Lyons et al., 1985 [5]	Clin Orthop Relat Res	II	Retrospective study	USA	Retrospective study evaluating plain radiographs on 50 confirmed painful hip arthroplasties. The aim was to identify radiographic findings of common complications	16	Two findings appear to be indicative regarding periprosthetic joint infection: scalloped endosteal resorption and laminated periosteal new bone. When scalloped resorption was present, it was associated with infection in 90% (47% sensitivity; 96% specificity). Laminated periosteal new bone occurred only in 25% of infected cases (25% sensitivity; 92% specificity)
Manrique et al., 2018 [54]	Arch Bone Jt Surg	II	Retrospective study	USA	A retrospective case–control study was performed with 56 patients undergoing septic revision arthroplasty and 112 patients undergoing aseptic revision arthroplasty. The aim was to identify risk factors for heterotopic ossifications	56	The incidence of heterotopic ossifications following surgical treatment of periprosthetic joint infection of the hip and aseptic revision of the hip was 84% and 11%, respectively. Cases after periprosthetic joint infection have significantly higher Brooker grades
Rajkumar et al., 2021 [46]	Indian J Orthop	IV	Retrospective study	India	Retrospective analysis on 66 primary and revision hip arthroplasties using a reconstruction ring. With an overall survival rate of 87% after 6.3 years and satisfactory functional outcome, the evaluation confirms the role of reconstruction rings in acetabular defects	2	In two cases after total hip arthroplasty with a reconstruction ring, a periprosthetic joint infection was present. Radiologically, loosening was seen on plain radiographs
Rey Fernández et al., 2021 [56]	J Bone Jt Infect	III	Case–control study	Spain	A case–control study with 26 patients that were diagnosed with periprosthetic joint infection after primary total hip replacement and 52 control patients without septic complications was conducted to evaluate the soft-tissue thickness (measured from trochanter to the skin) as a risk factor for periprosthetic joint infection	26	A strong association between higher soft-tissue thickness and risk for periprosthetic joint infection was observed. Patients with more than 60mm soft-tissue thickness had a sevenfold higher risk of infection
Riegler et al., 1976 [70]	Clin Orthop Relat Res	IV	Retrospective study	USA	A retrospective analysis of 102 patients undergoing total hip arthroplasty was performed. Plain radiographs, a physical examination as well as a questionnaire were evaluated. The aim was to develop a radiographic classification system of heterotopic bone formation and to find causative factors	2	Heterotopic ossification occurs in about 50% of all total hip arthroplasties. Only 2% develop severe heterotopic ossification. Only two patients out of the collective experienced a superficial infection and none of the patients had a deep periprosthetic joint infection. One of the two patients developed heterotopic ossifications whereas the other one had normal plain radiographs. Heterotopic ossification was also more likely in patients with postoperative hematomas and prolonged wound drainage
Ring, 1974 [48]	J Bone Joint Surg Br	IV	Retrospective study	UK	A retrospective analysis of 1000 hips with two different generations of implants (all implants were uncemented and had a metal-to-metal articulation) was conducted. Patients were assessed before the operation and one year postoperatively. Clinical outcome and complications were presented	7	Of the seven periprosthetic joint infections, only one had radiological changes. This case presented with an acetabular fracture which failed to unite
Rosteius et al., 2019 [60]	Arch Orthop Trauma Surg	II	Retrospective study	Germany	Analyses the frequency and potential risk factors to develop heterotopic ossification after PJI including 150 patients with a follow-up time of at least six months	150	70 patients (46.7%) developed heterotopic ossification after surgical care of PJI - Brooker I: 27 patients - Brooker II: 23 patients - Brooker III: 15 patients - Brooker IV: 5 patients Risk factors for developing HO after PJI are male gender, smoking, chronic infections and high number of previous operations
Schuldt et al., 2020 [49]	Nucl Med Commun	II	Retrospective study	Switzerland	Analyses the performance of radiography and CT scan in painful hip arthroplasty and their impact on the survival of the arthroplasty. 263 patients with painful hip arthroplasties were selected. With a median follow-up time of 41 months, pictures were counted as positive if they indicated the need of surgical revision	8	Out of eight patients with confirmed PJI, one showed loosening and suspicion of infection, three showed no signs of loosening or infection, three showed signs of cup/shaft loosening and one showed no signs of loosening
Sirka et al., 2016 [50]	Acta Orthop	II	Prospective study	Scandinavia	Prospective analysis of the Müller acetabular reinforcement ring (ARR) which was used for 321 primary arthroplasties with acetabular bone stock deficiency	6	Two of six septic loosening could be detected radiographically: - one showed radiological loosening and extensive medial and superior osteolysis - one showed osteolysis around the screws
Tapadiya et al., 1984 [51]	Clin Orthop Relat Res	II	Retrospective study	USA	Analyses the predictability of the outcome of total hip arthroplasty based on initial postoperative radiographs (*n =* 335) with a follow-up time of five years in average	5	Signs on radiographs suggestive for PJI: - cement mantle irregularities - subsidence of the femoral component
Tigges et al., 1994 [7]	AJR Am J Roentgenol	II	Retrospective study	USA	Analyses plain radiographic findings of 20 patients with confirmed infected hip prostheses	20	Radiographs of PJIs often show normal findings (10 out of 20 cases). Also nonfocal lucencies that usually are associated with mechanical loosening could be found. Other radiographic signs of PJI were focal bone loss that mimics aggressive granulomatosis, which could be found in two cases
Ure et al., 1998 [47]	J Bone Joint Surg Am	II	Prospective study	USA	Evaluates the outcome of single-stage hip arthroplasty in periprosthetic joint infections (*n =* 20) over a follow-up period of nine years	20	All 20 patients showed loosening of the prosthesis in plain radiographic images before revision. No further conventional parameters were reported

Heterotopic bone formation can occur in PJIs [53], but it can also be present in cases of aseptic loosening or just idiopathic [52]. In a retrospective study including 168 patients, Manrique et al. [54], reported an incidence of heterotopic ossifications following surgical treatment of PJI of the hip 84% and in aseptic revision cases of only 11%. Cases after periprosthetic joint infection in that study also had significantly higher Brooker grades [54]. In a CT-based study, Isern-Kebschull et al., reported a sensitivity for periarticular ossifications of 20% (8.4–36.9), a specificity of 79% (66.3–88.1), a positive predictive value of 35 (15.4–59.2), and a negative predictive value of 63 (51.3–73.9) [55].

Regarding risk factors for infection, an interesting observation was made by Rey Fernandez et al. who described that patients with more than 60 mm soft-tissue thickness over the greater trochanter had a sevenfold higher risk of infection [56].

Only one study explicitly elaborated on the detected bacteria and described the associated radiologic findings: Barrack et al. [44], reported 5 cases infected with *S. epidermidis* and found periostitis (3×), focal lysis (1×), diffuse lysis with endosteal scalloping (1×) and heterotopic ossification (2×). One case with *Strept. sanguis* had focal lyses around the implant.

## Discussion

PJI is one of the most serious complications after total hip arthroplasty and poses major diagnostic challenges for clinicians. Radiological imaging, especially plain radiographs, is part of every diagnostic workup in case of suspected complications after total hip arthroplasty. There are plenty of reviews and original works dealing with the diagnostic algorithm of PJI. This review aimed to summarize and analyse the available information about the radiologic presentation of PJI and the frequency of appearance. Furthermore, we raised the question of whether the causative microorganisms differ regarding the radiologic presentation.

The first observation of this review is the lack of original data evaluating the radiologic characteristics of PJI of the hip. There are more reviews (*n =* 26) repeating findings of other studies than original works (*n =* 18). These reviews mainly concentrate on the diagnosis of PJI, however, they do not focus on the radiographic presentation of PJIs. As described in the section "results", the main statements regarding the radiologic presentation in the reviews are the above-mentioned radiologic pathologies. They do not report on the frequency of the radiologic findings and no correlation to causative microorganisms was analysed. Furthermore, the findings regarding the radiologic presentation of PJI of original works are often coproducts of other primary endpoints [5, 44, 46, 48, 50, 51, 53].

The next interesting finding of this review is the frequency of radiologic abnormalities in the case of a proven PJI of the hip. While some authors report radiographic changes in all of their PJI cases (100%) [44, 45, 47], other authors only presented abnormalities in 14–63% [48–50, 57]. Multiple reasons could explain this wide range. Therefore, having a closer look at the original works reporting the frequencies is necessary. The first possible explanation is the lack of a standardized follow-up to classify radiologic changes in the case of PJI. Some authors present radiographs already 6 months after surgery while other authors describe X-rays taken nine years postoperatively. The given collectives are mostly very small with numbers of patients between 6 and 20. Such collectives are not big enough to give sufficient information about the frequency of the appearance of radiological abnormalities.

Furthermore, the oldest and latest works reporting frequencies are separated by more than 40 years of medical evolution [45, 47]. The definition and the diagnostic algorithm have, however, relevantly changed since then and therefore these studies lack comparability [58, 59]. Similarly, the technique of acquiring radiographs has also improved with the advent of digital radiography. This allows post-imaging optimization of each X-ray to visualize also structures such as soft tissues that were difficult to discern in traditional images intended for a good bone contrast only.

When using radiographs not only to exclude differential diagnoses, but also to diagnose a PJI, sensitivity and specificity are crucial. Two original works are reporting about the diagnostic value with similar results. The sensitivity could be classified as low at 20–25% while the specificity of periosteal reactions and periarticular calcifications is moderate to good (79–92%) [5, 52]. Consequently, plain radiographs could be used to confirm PJI in the presence of characteristic radiologic abnormalities. Especially in the case of early PJI, the majority of cases of early PJI radiographs are still usually normal [43]. As a consequence, the question of how to interpret and use radiographs to diagnose late PJIs and the diagnostic value of this technique remains equivocal with the analysed literature.

Having a look at radiographs taken after septic revisions of hip arthroplasties, three original works report heterotopic ossifications. Incidences range depending on the collective analysis between 12 and 84%. Brooker grades presented were mainly between one and three [53, 54, 60]. Interestingly, when comparing aseptic and septic revisions, heterotopic ossifications are significantly more likely in septic cases (11 vs. 84%) and present with significantly higher Brooker grades [54]. Consequently, some kind of interaction between soft tissue and the causing microorganisms can be assumed. This assumption is highlighted by the observation of Rey Fernández et al., that there is an association between higher soft-tissue thickness and risk for PJI after primary total hip replacements. Furthermore, patients with more than 60 mm soft-tissue thickness around the major trochanter had a sevenfold higher risk of PJI [56]. Unfortunately, there are no data available considering the association between the causing microorganisms and soft-tissue changes.

Historically and as presented in the analysed reviews, characteristics that are described to recognize a PJI of the hip on a plain radiograph are periosteal reaction, a wide band of radiolucency at the cement–bone or metal–bone interface, patchy osteolysis, implant loosening, bone resorption around the implant, and transcortical sinus tracts. In cases of aseptic loosening, there is slow and progressive evolution, while in cases of infectious loosening, this loosening occurs rapidly, in a more aggressive manner and with greater bone destruction [7, 61]. Data regarding the frequency of occurrence of the named radiologic abnormalities in cases of PJI are insufficient. Prospective or at least retrospective analyses with larger collectives are necessary to evaluate the frequency and to define the diagnostic value of the radiologic presentation for the diagnosis of PJI. Furthermore, there is a lack of data analysing the association between the causative microorganisms and the radiologic appearance. Although imaging techniques like computer tomography [52, 55, 62] or magnetic resonance imaging [63] have been reported to also provide additional and helpful clues concerning the presence of a PJI such as periosteal reaction, capsule oedema, and intramuscular oedema, the mainstay in diagnosing PJI to date remains plain radiographs.

## Conclusion

Typical radiologic abnormalities of PJI are periosteal reaction, a wide band of radiolucency at the cement–bone or metal–bone interface, patchy osteolysis, implant loosening, bone resorption around the implant, and transcortical sinus tracts. The frequency of their occurrence is still inadequately defined. A deeper understanding of the underlying causes and the relation to microorganisms can probably help clinicians in the future to diagnose a PJI. This is why further research should still further evaluate the radiographic features and value in the context of PJI.

## Data Availability

The datasets generated during and/or analysed during the current study are available throughout the manuscript.
